# Molecular Mechanisms Responsible for Increased Vulnerability of the Ageing Oocyte to Oxidative Damage

**DOI:** 10.1155/2017/4015874

**Published:** 2017-10-18

**Authors:** Bettina P. Mihalas, Kate A. Redgrove, Eileen A. McLaughlin, Brett Nixon

**Affiliations:** ^1^Priority Research Centre for Reproductive Science, School of Environmental and Life Sciences, University of Newcastle, Callaghan, NSW, Australia; ^2^School of Biological Sciences, University of Auckland, Auckland, New Zealand

## Abstract

In their midthirties, women experience a decline in fertility, coupled to a pronounced increase in the risk of aneuploidy, miscarriage, and birth defects. Although the aetiology of such pathologies are complex, a causative relationship between the age-related decline in oocyte quality and oxidative stress (OS) is now well established. What remains less certain are the molecular mechanisms governing the increased vulnerability of the aged oocyte to oxidative damage. In this review, we explore the reduced capacity of the ageing oocyte to mitigate macromolecular damage arising from oxidative insults and highlight the dramatic consequences for oocyte quality and female fertility. Indeed, while oocytes are typically endowed with a comprehensive suite of molecular mechanisms to moderate oxidative damage and thus ensure the fidelity of the germline, there is increasing recognition that the efficacy of such protective mechanisms undergoes an age-related decline. For instance, impaired reactive oxygen species metabolism, decreased DNA repair, reduced sensitivity of the spindle assembly checkpoint, and decreased capacity for protein repair and degradation collectively render the aged oocyte acutely vulnerable to OS and limits their capacity to recover from exposure to such insults. We also highlight the inadequacies of our current armoury of assisted reproductive technologies to combat age-related female infertility, emphasising the need for further research into mechanisms underpinning the functional deterioration of the ageing oocyte.

## 1. Introduction

The developmental potential of the mammalian oocyte markedly decreases with increasing maternal age, culminating in elevated rates of miscarriage, birth defects, and ultimately reduced fertility [1–4]. This loss of fecundity becomes evident when a woman reaches her midthirties. In particular, the incidence of chromosome abnormalities increases from approximately 2% for women in their twenties to 35% and 50% in their forties and fifties, respectively [3, 4]. Despite public misconceptions, current IVF technologies are unable to recover the fertility of older women with the live birthrate per oocyte steadily decreasing from 26% in younger women (<35) to just 1% for women at 42 [5]. The need to elucidate the mechanisms by which advanced maternal age negatively affects oocyte quality has become particularly pressing owing to the recent trend for women in developed countries to delay child bearing several years beyond that of their peak reproductive capacity. For example, in Australia, the average childbearing age increased from 27.7 years in 1987 to 30.7 years in 2008 [6]. In addition, the percentage of women having children later in life has also risen, with 8.5% of mothers being ≥35 in 1987 increasing to 24.4% in 2008 in Australia. Similar trends have also been observed in other developed countries including the UK, US, and Japan [6, 7].

More than two decades after being first proposed, the free radical theory of ageing remains a leading hypothesis to explain the deterioration of the ageing oocyte [8–11]. Indeed, an increase in intraovarian reactive oxygen species (ROS) has been convincingly correlated with increasing maternal age [12–15]. Moreover, several studies have drawn a compelling link between oxidative stress (OS) and the decline in oocyte quality [16–21] as well as *in vitro* fertilisation (IVF) and pregnancy success rates [15, 21–25]. The devastating consequences of OS on oocyte quality and female fertility have been comprehensively reviewed [9, 26–29]. Despite this, the molecular mechanisms that underpin the increased vulnerability of the aged oocyte to oxidative insults are still being elucidated. In this review, we provide a new perspective on reproductive ageing by exploring the underlying mechanisms behind the increased vulnerability of the ageing mammalian oocyte to OS. We consider the origin of elevated ROS in ageing oocytes but focus on the simultaneous decrease in the capacity of the oocyte to mitigate the detrimental impact of such oxidative insults. We also discuss the current means by which OS can be prevented or delayed in older mothers.

## 2. Overview of Oocyte and Follicular Development

**Figure 1:**
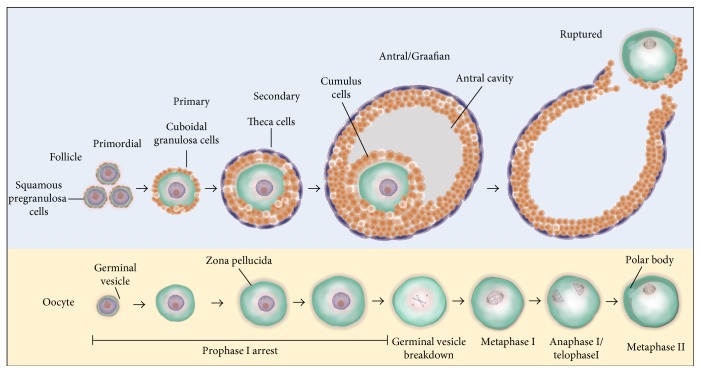
Stages of folliculogenesis and oocyte maturation. Primordial follicles consist of an immature GV oocyte arrested at prophase I, which is encapsulated by pregranulosa cells. Activation of primordial follicles to primary follicles is marked by a morphological change of pregranulosa cells from squamous to cuboidal. The development of the secondary follicle is marked by the acquisition of two or more layers of granulosa cells and the presence of a theca layer and contains an oocyte with a completely formed zona pellucida. The antral or Graafian follicle is the last stage of follicular development. This stage is marked by the presence of a follicular fluid-filled antral cavity adjacent to the oocyte. In the final stage of folliculogenesis, the oocyte achieves meiotic resumption, undergoing germinal vesical breakdown, and progresses through anaphase I and telophase I to complete meiosis I. At the completion of the first meiotic division, the first polar body is extruded and the ovulated oocyte becomes arrested once more at metaphase II until after fertilisation. Once the follicle is ruptured to release the mature oocyte, the remaining granulosa and theca cells differentiate into the corpus luteum.

The synergetic processes of folliculogenesis and oocyte maturation are required to produce oocytes capable of fertilisation. Primordial germ cells (PGCs) undergo mitotic proliferation to form a finite number of oogonia during prenatal life. In humans, folliculogenesis commences in utero in the second trimester with the recruitment of pregranulosa cells to the germ cells forming the primary functional unit of the ovary, the ovarian follicle [30] (Figure 1). These primordial follicles remain meiotically arrested in an extended prophase I, also known as germinal vesicle (GV) arrest, until they are recruited into the growing follicle pool for maturation and subsequent ovulation [31]. Upon activation, the primordial follicle experiences a period of follicle stimulating hormone- (FSH-) mediated follicular growth through primary, secondary, and antral follicle stages. This growth is accompanied by an accumulation of granulosa cell layers surrounding the oocyte, formation of the zona pellucida (ZP), and the differentiation of steroid-secreting theca cells at the basement membrane [30, 31]. Continual follicular growth sees the formation of the preovulatory follicle with the presence of an antral cavity containing plasma fluid and steroid hormones that are excreted by the granulosa cells, adjacent to the oocyte [32]. The oocytes populating preovulatory follicles remain in prophase I arrest yet have experienced significant growth, completed nuclear and cytoplasmic maturation, and are now surrounded by cumulus cells—a granulosa cell subtype [31]. A subsequent surge of luteinising hormone (LH) is responsible for initiating meiotic resumption and ovulation. During ovulation, the basement membrane of the follicle ruptures releasing a mature oocyte equipped for fertilisation [32]. The remaining granulosa and theca cells differentiate into the corpus luteum, which produces progesterone for pregnancy maintenance, or alternatively undergoes luteolysis [33]. Meanwhile, in the preovulatory follicle, the oocyte undergoes germinal vesicle breakdown (GVBD) and entry into the first meiotic division (MI) [34], during which chromosomes attach to the meiotic spindle and line up on the metaphase plate. Chromosomes then separate, with one pair of sister chromatids retained within the oocyte and the other extruded in the first polar body [35]. Following the first meiotic division, the oocyte becomes arrested once more, at metaphase II of meiosis (MII), whereupon transcription is temporarily repressed until after fertilisation at the 2-cell stage in mice or 4-cell stage in humans [33].

## 3. Aetiology of Elevated Cytosolic ROS in Ageing Oocytes

Traditional paradigms hold that the primary mechanism behind the age-related decline in oocyte quality is the accumulation of spontaneous damage to the mitochondria arising from ROS produced by the mitochondria themselves during daily biological metabolism [36]. Furthermore, there are several additional sources of ROS, attributed to either exogenous (i.e., lifestyle, stress conditions, and environmental factors) [37, 38] and/or endogenous factors (i.e., inflammation, cell proliferation, and apoptosis) [39], that can impact the ovarian environment and contribute to cellular ageing. While each of these factors have featured in elegant reviews [28, 40, 41], it has also recently been shown that the ovarian specific surges in ROS that accompany ovulation and luteolysis can act as a potent source of intraovarian OS with the potential to contribute to age-related decline in oocyte quality [20, 42]. Moreover, additional age-associated sources of ovarian ROS have been suggested to arise from the accelerated production of advanced glycation end-products (AGEs) and an accompanying decrease in the efficiency of perifollicular vascularisation [43, 44].

### 3.1. Ovulation-Induced ROS

Following FSH-mediated follicular maturation, an LH surge stimulates LH receptors on granulosa and cumulus cells, resulting in the generation of ROS and the concomitant depletion of antioxidant defences. This response is essential for promotion of granulosa cell apoptosis and breakdown of the follicular wall to permit oocyte release during ovulation [45–48]. The ROS generated have also been linked to integral roles in meiotic resumption via activation of the maturation-promoting factor (MPF) [48, 49]. Additionally, progesterone-induced OS is required for the induction of luteal cell apoptosis; a prerequisite for luteolysis with *in vitro* exposure of corpus luteum to ROS scavengers and antioxidants leading to a potent inhibition of this process [45, 50]. The potential damage arising from chronic exposure of oocytes to OS generated during recurrent cycles of stimulated ovulation has been highlighted in mouse models. Indeed, the induction of as few as three to six concurrent stimulated ovulatory cycles has been shown to elicit increased mitochondrial aggregation and mitochondrial DNA (mtDNA) mutations in oocytes, as well as oxidative damage to nuclear DNA, lipids, and proteins—lesions that collectively result in degenerative embryos and failure to reach blastulation [42]. Comparable phenotypes have also been documented after the induction of five sequential stimulated ovulation cycles in mice. In this study, it was determined that oocytes were of poorer functional quality and possessed significantly more mitochondrial defects, resulting in an accentuation of the level of intracellular OS [20]. Notably, these defects were ameliorated upon administration of the antioxidant L-carnitine throughout the repetitive ovulation cycles, indicating that repeated exposure to elevated ROS generated via folliculogenesis can certainly stimulate OS and precipitate a decline in oocyte quality [20]. In addition, repeated ovarian stimulation has been linked to progressive increases in spindle abnormalities, detached chromosomes, and cytoplasmic asters. Interestingly, these data were only observed after *in vivo* maturation (IVO), indicative of a compromised intrafollicular milieu [51]. In contrast, after four weeks of stimulation, an alternative mouse study reported a decline in meiotic competence during *in vitro* maturation (IVM), but not IVO. The latter was however associated with decreased ATP content in GV and IVO MII oocytes. Despite this, the authors failed to record any observable impact on implantation or reabsorption rates upon mating [52].

The injurious effects of repeated ovarian stimulation have also been demonstrated in several human studies of ovarian hyperstimulation, with consequential decreases in the frequency of implantation and pregnancy rates having been reported [53–55]. However, this evidence must be considered against that of other studies, which have failed to document any significant decline in ovarian response to repeated stimulation, including reports of no difference in the number of oocytes retrieved, embryo morphology, fertilisation and implantation, or pregnancy rates [56–59]. Despite the conflicting evidence emerging from human studies, it remains possible that oocytes from women of advanced maternal age, who will likely have experienced monthly ovulatory cycles for anywhere between three to four decades as well as a concomitant decrease in antioxidant defences (detailed below), are placed at heightened vulnerability upon exposure to additional source(s) of ROS.

### 3.2. Advanced Glycation End Products and Altered Vascularisation

An age-related increase in the levels of advanced glycation end products (AGEs) in the ovarian microenvironment has been newly postulated to contribute to ovarian ageing through the induction of elevated ROS [43, 44]. AGEs act to induce the generation of intracellular ROS by binding and activating ligand transmembrane receptors, known as RAGE (receptor for advanced glycation end products). Indeed, upon binding, RAGE triggers the downstream activation of NAD(P)H oxidase, mitogen-activated protein kinases (MAPKs), and the transcription factor nuclear factor kappa B (NF-*κ*B) [60–62]. This leads to an increase in intracellular ROS as well as upregulated expression of growth factors, cell-adhesion molecules, and proinflammatory cytokines and RAGE [63–67]. This positive feedback cycle ultimately culminates in a proinflammatory response and an increase in OS [64, 65, 68].

In support of the contribution of AGEs in ovarian ageing, Takeo et al. have recently reported significantly higher levels of AGEs in follicular fluid derived from aged cows when compared to their younger counterparts [14]. Furthermore, elevated levels of AGEs were associated with increased ROS as well as additional age-associated events including accelerated nuclear maturation, abnormal fertilisation, and decreased blastulation rates [14]. A corroborative study also reported an age-associated accumulation of AGEs and RAGE in human ovarian granulosa-lutein and monocytes [69]. Moreover, Tatone et al. documented reduced expression and activity of detoxifying methylglyoxal (an AGE precursor) in the ovaries of aged mice compared to those of young mice [70]. Taken together, these studies implicate AGEs as a contributor to the accelerating ROS generation observed in the ageing ovary.

Notably, AGEs have also been implicated in propagating age-related cellular damage in a more direct manner, independent of ROS induction, by inducing protein crosslinking [71]. Proteins with long half-lives such as collagen are the most vulnerable to this type of modification, leading to collateral damage in the form of altered vascular structure and function [72, 73]. Accordingly, it been suggested that the weakened efficiency of perifollicular vascularisation can be attributed to collagen damage induced by AGEs [43, 44]. Normal perifollicular vascularisation is essential to meet the demand for oxygen supply to the oocyte. This is particularly the case in advanced phases of follicular development where the oocyte relies on an ingrowth of capillaries into the surrounding theca cells [74]. It follows that impaired vascularisation resulting from inadequate capillary ingrowth can lead to a state of hypoxia, which in itself can trigger the generation of ROS and contribute to the pathology of age-related ovarian dysfunction [75]. The implications of inefficient vascularisation have been clinically demonstrated as oocytes derived from such follicles have reduced oxygen content (<3%) and lower fertilisation and developmental potential [76]. Accordingly, a negative correlation has been established between a woman's age and the degree of vascularisation observed in her late-stage ovarian follicles [77]. The converse is also true, whereby a positive correlation exists between highly vascularised oocytes and live birth rates resulting from IVF procedures [78, 79]. Further highlighting the potential significance of insufficient vascularisation in the aged ovary is the demonstration that oocytes retrieved from Graafian follicles with reduced vascularisation commonly present with spindle and chromosomal alterations similar to those witnessed in aged MII oocytes [80, 81].

### 3.3. Mitochondrial Defects

Several studies have converged on the notion that dysfunctional mitochondria represent the main source of elevated ROS within the ageing oocyte [36, 82]. Mitochondria hold central roles in calcium homeostasis, initiation of apoptosis, and cellular energetic metabolism within oocytes [83]. A key element of this metabolic strategy is the electron transport chain (ETC) that resides in the mitochondria and is responsible for the bulk of ATP generation within the oocyte; energy that is essential for the successful completion of meiosis [84]. Indeed, during spindle assembly in the MI and MII phases of oocyte maturation, mitochondria relocalise in dense clusters around the spindles in order to meet the enhanced demand for ATP [85]. This redistribution of mitochondria results in a burst of ATP production during oocyte maturation [86], the importance of which is highlighted by the fact that mitochondrial damage potently comprises GVBD, formation of the meiotic spindles, chromosome segregation, and polar body extrusion [87].

Mitochondria possess their own maternally transmitted multicopy genome (mtDNA) that acts independently of nuclear DNA. Oxidative damage to mtDNA is of particular importance as, unlike genomic DNA, these organelles lack protective histones and encompass limited mtDNA repair enzymes [88]. This underpins the hypothesis for why mtDNA have a 10- to 25-fold increased mutation and deletion rate relative to their nuclear DNA counterparts [89–91]. Accordingly, unfertilised oocytes retrieved from older women present with a higher incidence of chromosomal deletions and mtDNA point mutations [92, 93]. Such an increase in mtDNA mutations poses a significant threat to the health of the oocyte as it could lead to impairment of several mitochondrial encoded components of the ETC.

Maternal ageing has also been linked to mitochondrial dysfunction resulting in decreased oxidative phosphorylation and ATP generation [94, 95]. Indeed, global transcript analyses of human and mouse oocytes have revealed that a large proportion of age-related changes in transcript expression are associated with mitochondrial activity, including the downregulation of succinate dehydrogenase complex flavoprotein subunit A (*Sdha*) and genes coding for proteins associated with ATP production [3, 96, 97].

Additionally, Ben-Meir et al. demonstrated that generation of the coenzyme Q10 (CoQ_10_), which fulfils an essential role in the transport of electrons within the ETC, is compromised in the oocytes of ageing humans and mice. Furthermore, disruption of CoQ_10_ production elicits phenotypic changes that mimic the ageing effect in ovaries, with reduced ovarian reserve, decreased ATP production, and increased spindle abnormities resulting in infertility. Interestingly, oral administration of CoQ_10_ can reportedly reduce the level of the age-related decline in oocyte quality and quantity [98].

Similarly, Wilding et al. also reported that altered mitochondrial morphology and lower electron potential at the inner mitochondrial membrane in aged human oocytes was negatively correlated with the rate of embryo development [99]. Moreover, a decrease in mtDNA copy number has been recorded in bovine, mouse, hamster, and human oocytes with increasing maternal age and has been associated with a concomitant decrease in ATP production [93, 94, 100, 101]. In addition, oocyte mitochondria in aged mice and hamsters also exhibit altered morphology with vacuolisation, cristae alterations, and changes in cytoplasmic lamellae [94].

Among the most insidious consequences of mitochondrial dysfunction is an increase in ROS leakage from the ETC. Such elevated concentrations of ROS have the potential to set in train a cascade of events that culminate in a state of auto-oxidation whereby the mitochondria are unable to regulate the events of the ETC, thus exacerbating damage to mtDNA and proteins. During mitochondrial ATP production, ROS are released locally as a by-product, becoming a major source of intracellular ROS. Under normal physiological conditions, the mitochondria only reduce 0.1% of all oxygen entering the ETC into the prooxidant O_2_^·−^, which serves as a precursor to the majority of biological ROS [18, 102]. However, damage to the ETC has the potential to elevate O_2_^·−^ generation, leading to increased cellular oxidation. Studies from our research group have also established that in MII mouse oocytes, OS-catalyzed lipid peroxidation is capable of initiating cyclic ROS propagation via direct damage to mitochondrial components [103]. Specifically, we have shown that covalent modification of SDHA by the lipid aldehyde 4-hydroxynonenal (4-HNE) results in the auto-oxidation and subsequent transference of electrons to oxygen rather than reduction via CoQ_10_. This, in turn, leads to a decrease in mitochondrial membrane potential, an increase in OS, and the propagation of the lipid peroxidation cycle. As a consequence, oocytes experience DNA fragmentation and increased apoptosis [103]. Independent research has also established that exposure of mouse oocytes to exogenous H_2_O_2_ leads to the dissipation of mitochondrial membrane potential and a concomitant decrease in cytoplasmic ATP levels and the disassembly of MII spindles [104]. However, supplementation of the antioxidant *N*-acetylcysteine is able to ameliorate such damage [104].

## 4. Increased Vulnerability of the Ageing Oocyte to Oxidative Damage

### 4.1. Reduced Antioxidant Capacity and ROS Metabolism

Mammalian cells are endowed with a wide array of antioxidants with the ability to scavenge ROS, including nonenzymatic antioxidants such as vitamins A, C, and E and glutathione (GSH) as well as several enzymatic antioxidants including superoxide dismutase (SOD), glutathione peroxidase (GPX), catalase (CAT), glutathione S-transferase (GST), peroxiredoxin (PRDX), and thioredoxin (TXN) [105–110]. Enhanced levels of cellular OS with ageing have been, at least partially, attributed to the weakening of the antioxidant enzymatic defences present within the cell [111]. In combination with age-associated increases in prooxidants, it is likely that this scenario may enable free radicals to evade cellular defences and subsequently cause damage to a suite of macromolecules that are required to maintain oocyte viability. While reports of the impact of ageing on the expression and activity of various antioxidants are plagued by considerable variability, there is a general consensus amongst these studies that dysregulation of ROS metabolism is a feature of the ageing ovary and oocyte (Tables 1 and 2).

Antioxidant enzymes within granulosa cells, cumulus cells, and follicular fluid each play a critical role in the protection of the oocyte, owing to their ability to facilitate the scavenging of ROS, particularly during steroid hormone synthesis. Of concern, however, is that age-dependent dysregulation of antioxidant enzyme activity and/or expression has been reported in every one of these cellular compartments. For instance, a study of patients undergoing IVF reported lower gene and protein expression of SOD1, SOD2, and CAT in cultured granulosa cells from 38- to 42-year-old IVF patients compared to equivalent cells obtained from a younger patient cohort aged between 27 and 32 years [112] (Tables 1 and 2). Total SOD activity, as well as SOD1 protein expression, has also been shown to decrease within the cumulus cells surrounding ovulated oocytes from IVF patients of advanced maternal age. Moreover, such changes have been correlated with unsuccessful IVF outcomes [113]. In human follicular fluid, Carbone et al. observed a decrease in the levels of GST and CAT activities, higher SOD activity, and a decrease in GST protein expression in IVF patients aged between 39 and 45 years compared to younger patients aged between 27 and 32 years [114]. Decreased activity of GST, glutathione reductase (GR), and GPX is also characteristic of the follicular fluid recovered from poor IVF responders and has, in turn, been positivity correlated with elevated nitric oxide and the lipid peroxidation products of malondialdehyde (MDA) and 4-HNE [24].

Differing ovarian gene expression levels of critical antioxidant enzymes have also been documented in aged versus young mice and attributed to notable increases in lipid, protein, and DNA oxidation. This ageing phenomenon is true for both ovarian interstitial cells and the follicles themselves [13]. For instance, the expression of the cytosolic antioxidant *Gpx1* has been documented to increase in aged mouse ovaries, while the expression of glutathione *S*-transferase mu 2 (*Gstm2*) apparently decreases in this tissue. Additionally, the mitochondrial antioxidants *Prdx3* and *Txn2* also experience decreased expression in such models [13]. Similarly, significant attenuation of the activity of both SOD and GPX has been recorded in ovarian homogenates from premenopausal to menopausal women [115].

Global gene expression analysis of aged oocytes also revealed reduced expression of *Sod1* and *Txn* family members in ovulated mouse and human oocytes [3, 96, 97]. At the protein level, only modest decreases have been detected in the expression of GSTM5 and TXN1 in MII oocytes recovered from mature versus aged mice [116]. Additionally, the activities of GST and thiols have been reported to decrease in aged MII mouse oocytes [117]. In alternative model species such as the pig, chemical inhibition of SOD activity in oocytes has been shown to elicit a reduction in meiotic progression, decreased GSH levels, and diminished rates of cleavage and blastocyst formation [118]. Such defects have also been documented in the hamster and bovine, where the depletion of GSH has been associated with altered spindle morphology, disturbed microtubule function, and chromosome clumping in MII oocytes [119, 120].

More recently, the hormone melatonin, which has strong antioxidant capacity, has also been shown to be downregulated upon ageing. Indeed, reduced levels of melatonin have been recorded in the follicular fluid of women of advanced maternal age [121]. The authors of these studies also reported a positive correlation between melatonin levels and IVF outcomes, with higher ovarian reserves, numbers of collected oocytes, fertilised oocytes, cleaved zygotes, high-quality embryos, blastocysts, and embryos suitable for transplantation all being documented in parallel with elevated melatonin. Accordingly, an inverse correlation has been established between lower levels of melatonin and higher levels of the lipid peroxidation product MDA in the serum obtained from infertile female patients [122].

### 4.2. Downregulation of Sirtuin Proteins

The sirtuin (SIRT) family of proteins have been newly established as having strong antioxidant capacity, an important biochemical property in the context of the protection they afford to oocytes from oxidative insults [123]. SIRT proteins possess either NAD^+^-dependent deacetylases or mono-ADP-ribosyltransferase activity and have been shown to modulate ageing and cell metabolism, primarily by guarding cells against the damaging impact of oxidative insults [123–125]. SIRT proteins have proven to be essential in oocytes, with inhibition of total SIRT activity resulting in the disruption of meiotic maturation, the formation of the actin cap and the cortical granule-free domain, and induced spindle defects and chromosome misalignments [126].

A decrease in SIRT1 protein expression and an increase in *Sirt1* gene expression have been observed in GV oocytes of aged mice [127]. Di Emidio et al. also detected a relocalisation of SIRT1 and the upregulation of *Sirt1* expression in response to the induction of OS in mouse GV oocytes. These changes occurred commensurate with a decrease in *miR-132*, a microRNA implicated in the posttranscriptional regulation of the *Sirt1* transcript. Interestingly, aged oocytes were found to be characterised by a lower basal expression of *miR-132* and, upon exposure to oxidative insults, did not experience a further reduction in *miR-132* or concomitant increase in *Sirt1*, equivalent to the response recorded in the oocytes of younger females. Together, these data suggest that this stress response mechanism is compromised during maternal ageing. The authors of this study also reported a dose-dependent increase in intracellular ROS and a decrease in the number of oocytes reaching the MII phase of development, upon inhibition of SIRT1 activity [127]. SIRT1 inhibition also prevented the upregulation of the antioxidant *Sod2*, in response to OS, indicating that the protein likely acts upstream to mediate *Sod2* gene expression. In additional studies, an age-dependent decrease in SIRT1 protein expression has been linked to chromatin disorganisation in the GV oocyte, a defect possibly arising from an inability to modulate the activity of histone methyl-transferase and subsequent trimethylation of histones [128, 129].

Remarkably, transgenic mice engineered to overexpress SIRT1 presented with a pronounced delay in reproductive ageing accompanied by a decreased time to conception compared to that of wild-type control mice [130]. Similarly, resveratrol-mediated upregulation of SIRT1 in IVM bovine oocytes has resulted in an improved fertilisation rate and blastocyst numbers and increased mtDNA copy number, membrane potential, and ATP content in the mature oocyte [131]. Furthermore, a rapamycin-mediated elevation of SIRT1 and SIRT6 expression in rat ovaries has also been associated with preservation of the primordial follicle pool [132]. Overall, these studies suggest that a SIRT1-mediated decrease in ROS may contribute to the preservation of fertility under conditions of ageing and oxidative stress.

In addition to SIRT1, the SIRT3 isoform also has distinct roles in the deacetylation and activation of diverse mitochondrial enzymes involved in antioxidant protection (e.g., glutamate dehydrogenase 1), the metabolism of amino acids and fatty acids, and in the electron transport chain [133, 134]. Indeed, an age-dependent decrease in SIRT3 gene and protein expression in human granulosa and cumulus cells of IVF patients has been associated with attenuation of GSH deacetylation, mitochondrial activation, and altered ROS metabolism, thus contributing to a depleted ovarian reserve [133]. Moreover, pan SIRT inhibition, siRNA-induced knockdown of *Sirt3* in fertilised eggs, and the targeted ablation of *Sirt3* in global knockout mouse models have each been shown to increase mitochondrial ROS production and repress blastulation and postimplantation development in mouse embryos generated via IVF [135]. Interestingly, the same study also reported an upregulation of SIRT3 protein and *Sirt3* mRNA expression in response to OS. Moreover, under conditions of low oxygen, 2-cell embryo and blastocyst formation were unaffected by siRNA knock down, with ROS levels also remaining low [135]. These findings highlight the importance of SIRT3 activity under conditions that lead to an induction of OS.

In terms of alternative isoforms of the sirtuin family, the expression of both *Sirt2* and *Sirt6* has also been shown to decrease in the cumulus cells of aged mice, suggesting they too may contribute to the hierarchy of mechanisms that protect the quality of oocytes in young animals [136]. Accordingly, SIRT2 depletion in mouse oocytes has been linked to spindle defects, chromosome disorganisation, and impaired microtubule-kinetochore interactions [137]. A similar spectrum of lesions (i.e., spindle defects, chromosome misalignment, impaired kinetochore-microtubule interactions, and aneuploidy during meiosis) has also been reported in the oocytes of mice targeted for *Sirt6* knockdown [138]. Conversely, a range of age-related oocyte pathologies can be mitigated via the overexpression of SIRT2 in these cells [137]. Taken together, these data highlight the important role that several members of the SIRT family of proteins hold in protecting the oocyte from oxidative stress and regulating oocyte meiotic events, and the detrimental pathologies that develop if these proteins are downregulated during the ageing process.

### 4.3. Compromised Oxidative DNA Damage Repair Pathways

An additional characteristic of the aged oocyte that renders them particularly vulnerable to oxidative attack is the downregulation of genes involved in DNA damage repair. DNA damage can result in alterations in gene expression mediated through epigenetic modifications and mutagenesis [139–141]. Oocytes are acutely susceptible to accumulating DNA damage due to their extended prophase arrest. Indeed, an increase in DNA double stand breaks (DSBs) has been detected in aged mouse and human primordial follicles as well as in GV oocytes [142–144]. Alternatively, the interstitial tissue of ageing mouse ovaries has also been shown to accumulate a higher proportion of oxidative DNA damage, detected via measurement of the DNA oxidation marker 8-hydroxy-2′-deoxyguanosine (8-OHdG), than that of an equivalent tissue in young animals [13]. This is particularly concerning as elevated intrafollicular levels of 8-OHdG lesions positively correlate with high rates of degenerative oocytes in women [21]. The causative nature of these phenotypes is suggested by evidence that antioxidant treatments aimed at ameliorating 8-OHdG concentrations can enhance a woman's chances of conceiving and maintaining pregnancy, as has been documented amongst a cohort of women that had previously experienced failed pregnancies arising from IVF embryo transfers [21]. In rat models, it has been shown that GV oocytes subjected to exogenous H_2_O_2_ experience an increase in DNA fragmentation and eventually succumb to apoptosis [17]. Studies of this nature serve to illustrate the pervasive nature OS and the capacity of this insult to elicit DNA damage and compromise the fidelity of the ageing oocyte.

Such findings take on added significance in view of the fact that the efficacy of DNA DSB repair mechanisms become attenuated in aged oocytes. Indeed, an increase in the expression of the DNA DSB damage maker *γ*H2AX in the primordial follicles and GV oocytes from aged mice and humans correlates with a decline in the expression of several DNA DSB repair genes including, *Brca1*, *Mre11*, *ATM*, and *Rad51* [142]. The decrease in *Brca1* expression in the MII oocytes of aged mice was also observed by Pan et al. with the RNAi-mediated reduction of *Brca1* resulting in abnormal spindle formation, chromosome misalignment, and a significant increase in hyperploid oocytes [3]. Interestingly, microarray analysis of human MII oocytes recovered from aged versus young donors has also revealed that the former are characterised by an apparent decrease in several genes associated with the DNA damage checkpoint, including *Atr*, *Chek1*, *Nbs1*, and *Rad17* [96]. The importance of oxidative DNA damage repair during ageing has been further highlighted in senescence-accelerated mice (SAM) models. Using this mouse strain, it has been demonstrated that increased oxidative damage brought about by mutations in mtDNA and the oxidative DNA repair enzyme OGG1 leads to accelerated ageing phenotypes including spindle and chromosomal abnormalities [145, 146]. While the consequential reduction in DNA repair capacity argues that oxidative insults could elicit a higher level of DNA damage in an aged oocyte, this has yet to be experimentally confirmed.

Aside from DNA damage, telomere shortening has also been documented as a consequence of both ageing and OS in the ovarian environment [147, 148]. Telomeres are comprised of repetitive DNA nucleotide sequences and associated proteins localised to the end of eukaryotic chromosomes. These entities serve a predominantly protective function to maintain chromosomal integrity and prevent end-to-end chromosome binding [149]. During ageing, telomere length gradually shortens due to repeated cycles of DNA replication and the adverse effects of a variety of genotoxic agents including OS [150]. Although oocytes and their surrounding granulosa cells are among a limited number of normal adult cell populations endowed with telomerase enzymes to counteract telomere shortening and ensure genetic stability, the telomerase activity of these ovarian cells is reduced in women of advanced maternal age [147, 151]. Similarly, in aged mouse models, oocytes experience a reduction in gene and protein expression of telomerase reverse transcriptase (TERT), a catalytic subunit of telomerase, as well as a consequential attenuation of telomerase activity [148]. Telomeres are a primary target of DNA damage in ageing human somatic cells, and severely shortened or uncapped telomeres result in genetic instability and cellular senescence [152]. By analogy, telomere shortening in oocytes during reproductive ageing may also predict developmental competence.

### 4.4. Reduced Fidelity of the Spindle Assembly Checkpoint

Stringent molecular mechanisms exist to prevent oocytes with significant DNA damage from progressing through meiosis and/or embryo development. Damage to genomic content of primordial and primary follicles ultimately leads to the induction of an apoptotic cascade via activation of the transcription factor transformation-related protein 63 (TAP63). However, oocytes that have proceeded to secondary and more advanced stages of development fail to express TAP63 and are therefore reliant on the meiotic spindle assembly checkpoint (SAC) to halt the development of any cells compromised by DNA damage and/or chromosomal abnormalities [153, 154]. In oocytes harbouring DNA damage induced by chemical agents and UV radiation, SAC activity increases resulting in MI arrest [155–157].

Despite this checkpoint, in ageing mothers, an increase in DNA damage and abnormal MII oocytes and embryos is more likely to occur than in their younger counterparts. This is consistent with evidence that the fidelity of SAC is compromised in aged oocytes, leading to a situation in which aged oocytes harbouring DNA damage are able to more readily evade MI arrest [3, 155]. This suggests that SAC failure is a likely contributor to the increased incidence of chromosome abnormalities documented in oocytes and embryos from older women. Concomitantly, there is mounting evidence from somatic cells that even modest concentrations of H_2_O_2_ can compromise the stringency of the SAC [158]. By analogy, it is tempting to speculate that oxidative induced DNA damage, which escapes the SAC in aged mothers, could increase the incidence of chromosomal defects in oocytes and embryos.

### 4.5. Downregulation of Reversible Protein Oxidation Repair

Oocytes are also equipped with stringent repair and proteolytic pathways to mitigate the impact of oxidatively damaged proteins. Upon oxidation, targeted proteins are subject to repair or, in the event that the damage is too great to mount complete repair, they are selectively degraded [159, 160]. Unfortunately, only limited oxidative-based modifications of the sulfur-containing amino acids, cysteine, and methionine possess the capacity for reversibility via reduction back to their native form [161, 162]. For instance, oxidation of the sulfhydryl groups in cysteine can be reduced by the thioredoxin-thioredoxin reductase system and glutaredoxin-glutathione-glutathione reductase system. Glutaredoxin and thioredoxin reduce disulfide bonds of cysteine by acting as electron doners and are subsequently reduced by glutathione-glutathione reductase and thioredoxin reductase, respectively, in an NADPH-dependent manner. An alternative class of enzymes, the methionine sulfoxide reductases (MSRS), are responsible for the conversion of methionine sulfoxides back to methionine, with the resulting sulfenic acid intermediate being reduced by the thioredoxin-thioredoxin reductase system [162, 163].

The importance of a fully functioning thioredoxin-thioredoxin reductase system is further emphasised by the fact that homozygous KO of either *Txn1* or *Txn2* is embryonically lethal, with cells derived from the inner cell mass of *Txn1* KO animals unable to proliferate [164, 165]. It is therefore of concern that the gene expression of the thioredoxin family has been shown to decease in the ovaries and oocytes of aged mice. Indeed, members of the thioredoxin family such as *Txn1* and thioredoxin domain-containing protein 9 (*Txndc9)* are each characterised by decreased expression in the MII oocytes of 42- to 45-week-old mice compared to that of younger, 5- to 6-week-old animals [97]. Similar reduced expression profiles have also been reported for thioredoxin reductase 1 (*Txnrd1*) and *Txnrd3* in the GV and MII oocytes of 66-week-old mice versus that of 6-week-old animals [3]. This trend also applies to *Txn2*, which experiences a notable decrease in ovarian expression between 2- and 12-month-old mice [13]. Consequently, a marked decrease in reduced sulfhydryl groups, indicative of an elevated level of protein oxidation, has been observed in follicular fluid recovered from aged women [43]. Taken together, these data indicate that the capacity to repair reversibly oxidised proteins may be compromised in the aged oocyte owing to the downregulation of the reductase systems tasked with this role, which could manifest in catastrophic consequences for the oocyte.

### 4.6. Compromised Degradation of Irreversible Protein Oxidation

**Figure 2:**
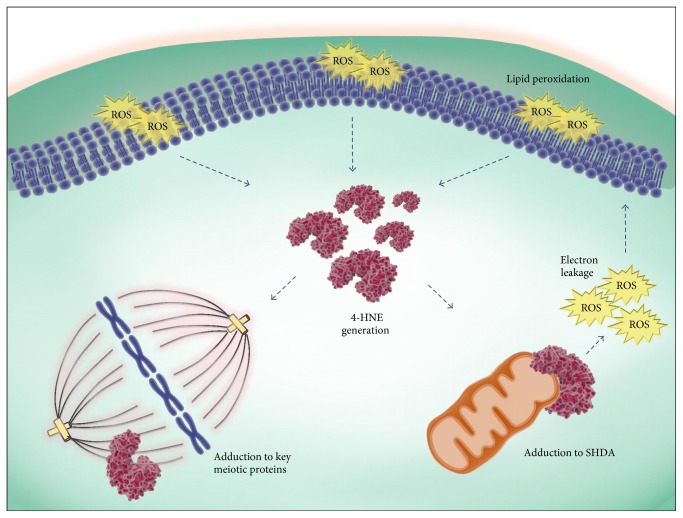
Cyclic propagation OS via lipid peroxidation impacts oocyte quality. Upon the induction of OS, ROS can instigate the peroxidation of lipids and the subsequent generation of highly electrophilic lipid aldehyde by-products such as 4-HNE. 4-HNE has the capacity to covalently modify and damage a wide array of proteins, including those essential for meiosis [174]. Additionally, adduction of mitochondrial SDHA impairs the ETC chain leading to electron leakage and the initiation of a positive feedback loop resulting in the generation of more ROS and lipid aldehydes [103].

In contrast to the situation described in the previous section, the majority of nonenzymatic oxidative protein modifications are actually irreversible, a situation that prevents their repair and instead targets the damaged proteins for proteolysis [166, 167]. In terms of the nature of these modifying agents, it is well-established that they include a variety of highly reactive aldehyde species that are formed as secondary products of the lipid peroxidation cycle, including 4-HNE (Figure 2()) [168–172]. Indeed, an elevation in the levels of 4-HNE has been demonstrated in whole ovary and the interstitial tissue of aged mice ovaries [13, 173]. Similarly, in recent work conducted in our laboratory, this oxidative lesion also appeared to be concentrated in the vicinity of the oocyte [174]. Accordingly, we identified a significant increase in the level of 4-HNE-modified proteins in GV and MII stage mouse oocytes between 4–6 weeks and 14 months of age [174]. Moreover, direct exposure of oocytes from these young mice to exogenous 4-HNE resulted in a range of defects that mirrored those witnessed in aged oocytes. Such changes included decreased meiotic completion, increased spindle abnormities, chromosome misalignments, and elevated aneuploidy rates. Our study also identified *α*-, *β*-, and *γ*-tubulin as major targets for 4-HNE modifications in both aged oocytes as well as young oocytes exposed to exogenous oxidative insult in the form of either H_2_O_2_ or 4-HNE. On the basis of these data, we infer that the susceptibility of key meiotic proteins, such as tubulins, to 4-HNE modification contributes to the increased aneuploidy rates recorded in aged oocytes.

In addition to tubulin, several other functionally important proteins have also been shown to be vulnerable to oxidative adduction by reactive aldehyde species. In the case of mouse oocytes, previous work from our laboratory has identified SDHA, a component of complex II of the mitochondrial electron transport chain, as a proven target for adduction by 4-HNE, and possibly MDA and acrolein, thus further implicating these reactive aldehydes in the deteriorating quality of the postovulatory aged eggs [103] (Figure 2()). Interestingly, both alpha-tubulin and SDHA appear to be conserved targets for 4-HNE adduction in alternative cell types such as the male gamete [175, 176]. However, additional targets in these cells are known to include the molecular chaperone, HSPA2, which facilitates ZP binding, as well as dynein heavy chain, cAMP-dependent protein kinase, alpha-catalytic subunit (PRKACA), and tubulin polymerisation-promoting protein family member 2 (TPPP2), each of which are each important for sperm functionally [175–177]. The detrimental impact of each of these phenomena on cellular proteostasis highlights the impetus for rapid clearance of the recipients of oxidative damage.

**Figure 3:**
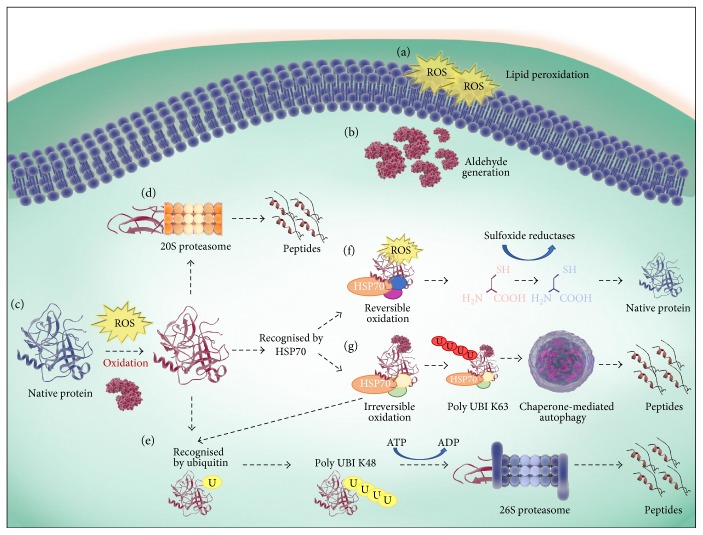
Repair and degradation mechanisms of oxidised proteins. (a) Elevated ROS induces a state of OS resulting in the peroxidation of lipids and (b) the generation of lipid aldehyde by-products such as 4-HNE. (c) During OS, native proteins can be oxidised directly by ROS or by secondary by-products of oxidation, such as 4-HNE. There are several pathways for the resolution of oxidised proteins; (d) oxidised protein can be degraded into peptides directly by the proteasomes 20S catalytic core, or (e) can be modified by ubiquitin and polyubiquitinated via K48 to be recognised and degraded by the 26S proteasome in an ATP-dependent manner. Alternatively, the oxidised protein can also be recognised by HSP70s. (f) In the case of revisable oxidation, HSP70s, in combination with cochaperones, mediate protein reduction and refolding back to their native form. (g) Where HSP70 recognition occurs and the oxidative modification is irreversible, such as 4-HNE adduction, the HSP70 and an alternate subset of cochaperones act to facilitate protein degradation via mediating polyubiquitination via K63 (recognised by the autophagy machinery) or K48 (recognised by the 26 proteasome).

The degradation of irreversibly damaged protein is most often mediated by the ubiquitin-proteasome system (UPS) [178–180]. In the UPS, proteins are covalently modified with multiubiquitin chains for targeted degradation by the 26S proteasome holoenzyme. This enzyme comprises a 19S regulatory subunit and a 20S catalytic core, each of which are composed of multiple subunits. In its regulatory capacity, the 19S subunit is responsible for controlling the entrance of ubiquitin-tagged proteins into the 20S core as well as the removal of the ubiquitinated chains thereby permitting target protein degradation by the 20S core. Despite this gate-keeper role, there is accumulating evidence to suggest that the predominant pathway utilised for the removal of oxidatively damaged proteins is via the 20S proteasome alone, independent of the 19S subunit, ATP, and/or ubiquitin [179, 180] (Figure 3).

In cells functioning under normal physiological parameters, the activity of the proteasome is upregulated in response to OS in order to resolve concomitant increases in dysfunctional and/or misfolded proteins [181–184]. Nevertheless, it has been convincingly demonstrated that the proteasomal system is generally less active in ageing somatic cells, thus decreasing the rate of oxidised protein resolution and creating an imbalance between ROS production and ROS clearance, factors that ultimately contribute to increased aggregate formation and accentuation of cellular damage [185–187]. Furthermore, this imbalance leads to a decrease in the ability of the ageing cell to prevail through oxidative attack.

Though it has yet to be proven directly, the activity of the proteasome is also likely to be downregulated in ageing oocytes owing to a decrease in transcript expression of several constituents of the UPS as detected via global comparative transcript analyses conducted on young versus aged mouse oocytes (GV and MII). Candidate genes identified in these studies included those encoding ubiquitin-activating enzymes (*Ube1c*), ubiquitin-conjugating enzymes (*Hip2*, *Ube2a*, *Ube2e3*, and *Ube2g1*), ubiquitin-ligases (*Siah2*), and ubiquitin itself (*Ubc*), as well other ubiquitination promoting enzymes (*Anapc4*). Proteasomal components appear to be equally susceptible to age-dependent decline, with documentation of a downregulation of transcripts encoding for 20S proteasome subunits (*Psma6*, *Psmb1*, *Psmb2*, *Psmb4*, and *Psmb5*), ATPase (*Psmc2*, *Psmc3*, and *Psmc5*), as well as the non-ATPase subunits of the 19S regulator (*Psmd4*, *Psmd8*, *Psmd9*, and *Psmd12*) [3, 97] (Table 1). Moreover, several of these transcripts appear to be similarly dysregulated in the MII oocytes of aged human donors [96] (Table 1). Although not as comprehensive, an equivalent decrease in the levels of proteasomal proteins, such as PMSD12, has also been detected in the MII oocytes of aged mice [116].

In contrast to this hypothesis, Tsakiri et al. demonstrated that the gonads and maturing oocytes of naturally aged *D. melanogaster* retain relatively high activity of the 26S proteasome and consequently accumulate less oxidatively damaged proteins than those of equivalently aged somatic tissues [187]. In addition, a study in *C. elegans* revealed that carbonylated proteins are abruptly eliminated by the proteasome in maturing oocytes, irrespective of the age of the mother [188]. While it is difficult to refute the possibility that such conflicting data may simply reflect species-specific responses, it must also be considered that the influence of ageing on proteasome activity does vary between distinct cell types. Indeed, the maintenance of proteasome activity within the oocyte may very well act as a compensatory mechanism to repair and protect against damage to the germline. Considering this, investigation into the existence of such potential compensatory mechanisms in mammalian ovarian tissue is clearly required.

Notwithstanding the obvious importance of the UPS, it has been suggested that the proteasome is only capable of degrading mildly oxidised proteins stemming from the fact that proteins must have the capacity to be unfolded prior to entering the relatively narrow 20S catalytic core [189]. Thus, the degradation of moderately to severely oxidised proteins that are unable to be sufficiently linearized rests with alternative lysosomal-dependent pathways [189]. Indeed, through the use of inhibitors capable of selectively, and independently, targeting the proteasomal or lysosomal pathways, it has been demonstrated in somatic systems that proteins bearing more extensive modifications are preferentially directed away from the proteasome and toward lysosomal degradation pathways whereupon they are recycled via the action of acidic lysosomal hydrolases [190]. This pathway has been demonstrated in *Xenopus* oocytes wherein lysosomal activity was stimulated upon intracellular accumulation of damaged proteins [191].

The lysosomal pathways can, in turn, act via direct engulfment of the substrate by the lysosomal membrane, or alternatively, substrates can be delivered to the lysosome by either molecular chaperone proteins or by a double membraned autophagosome that forms around cytoplasmic material [192, 193]. The latter catabolic mechanism, also known as autophagy, serves to compensate for the impairment of UPS activity under high oxidative burden [194, 195]. Interestingly, autophagy was shown to be upregulated in oocytes obtained from cows aged between 25 and 167 months, potentially suggesting that autophagic compensation may indeed be occurring in the context of the ageing oocyte. Curiously, chemical stimulation of autophagy in aged oocytes has been associated with an increase in oocyte quality [196].

Ubiquitination has also been implicated in coordinating the catabolism of proteins via selective autophagy, thus acting as a unifying factor linking the UPS, autophagy, and lysosomal pathways of degradation [197]. Accordingly, linker molecules exist with the capacity for interaction between both ubiquitin and components of the autophagy machinery. Indeed, under situations in which a cell's proteasomal capacity is overwhelmed by an accumulation of misfolded proteins, polyubiquitinated proteins tend to aggregate into large-scale aggresomes, which are subsequently degraded by autophagy. The formation of these aggresomes is critical for cell survival owing to their ability to mitigate the cytotoxic effects of free-floating misfolded proteins [198]. Illustrative of this process, the adaptor molecules HDAC6 and p62 have the capacity to bind mono and polyubiquitinated proteins and subsequently translocate them to aggresomes and to autophagic machinery, respectively [199–202]. Taken together, it is tempting to speculate that the dysregulation of ubiquitin and ubiquitin-related enzymes in the ageing oocyte might also compromise the efficiency of aggresome formation and the selective autophagy of oxidised proteins in oocytes.

### 4.7. The Role of Molecular Chaperones

Molecular chaperones are defined by their ability to confer resistance to environmental stressors and as such are often referred to the cell stress response or heat shock proteins (HSPs) [203, 204]. HSPs act by transiently associating with client proteins to mediate conformational stabilisation and aggregate prevention, relocalisation, or degradation [205]. The decision as to whether a protein will undergo protein repair and refolding or proteolysis is regulated in part by HSPs and their cochaperones. Indeed, it has been suggested that the HSP70 and HSP90 chaperone families and ubiquitin compete for the binding of a substrate, with those interactions favouring substrate-ubiquitin adhesion resulting in proteolysis [206]. In contrast, substrate-chaperone interactions can direct proteins towards either refolding and repair or proteolysis, with the protein's fate being largely arbitrated by cochaperones. Interactions with cochaperones that promote client binding and/or ATPase activity often facilitate protein repair and refolding [206, 207]. However, in situations where the substrate is unable to resume its folded conformation, chaperones can maintain the folded intermediate in a soluble form for recognition and catabolism by proteolytic machinery [208]. Conversely, interaction between the HSP70-HSP90 complex and the cochaperones BAG1 and CHIP, which can also act as an E3 ubiquitin ligase to catalyse the transfer of ubiquitin to the protein substrate, results in the inactivation of the HSPs ATPase folding activity, leading to client proteins being ubiquitinated and directed towards proteasomal degradation [206, 209].

The importance of HSPs in maintaining proteostasis within oocytes takes on added significance in light of evidence that oocytes obtained from aged mice exhibit a marked decrease in the gene expression of Hsp40s (*Dnaja2*, *Dnaja4*, *Dnajb1*, *Dnajb6*, *Dnajb10*, *Dnajb11*, *Dnajc3*, *Dnajc5*, *Dnajc8*, and *Dnajc21*), Hsp70s (*Hspa1b*, *Hspa4*, *Hspa8*, *Hspa9a*, *Hspa14*, and *Hsp70–3*), Hsp90s (*Hspcb*), and components of the chaperonin containing TCP1 complex (*Cct1–3* and *Cct5*) (Table 1) [3, 97]. Such reductions are particularly concerning given the vital role that HSPs play in survival and recovery from oxidative stress. Accordingly, several studies have reported an increase in gene and protein expression of HSPs in response to oxidative stress [207, 210, 211]. Conversely, it has also been shown that oxidative stress can impair the heat shock response, thus delaying resolution of unfolded proteins [212]. HSP70-mediated autophagy has also been demonstrated to be induced during oxidative stress and shown to accelerate the degradation of specific oxidized proteins [213, 214]. Moreover, HSP70 proteins have been shown to mediate dissociation and reassociation of the 26S proteasome during adaptation to oxidative stress [215]. Furthermore, HSP70 and HSP90 are known to interact with misfolded proteins to avert aggregation and initiate substrate refolding in an ATP- and cochaperone-dependent manner [216] with an elevation of HSP70 resulting in reduced aggregate formation via proteasome stimulation [217]. Ultimately, an age-related deterioration in the capacity of the oocyte to produce active heat shock proteins could contribute to an accumulation of oxidatively damaged proteins and their aggregation in ageing oocytes.

## 5. Therapeutic Interventions to Combat Age-Associated Decline in Oocyte Quality

### 5.1. Antioxidant Treatments

In recognition of the fundamental role that OS holds in the aetiology of ageing, in the following studies, the administration of antioxidant therapies has proven successful in improving both the quality and quantity of oocytes recovered from aged mice. In this context, potent antioxidants such as resveratrol have been successful in counteracting the ageing phenotype in mouse ovaries. Indeed, 12 months of resveratrol treatment, initiated at the time of weaning, resulted in an elevated follicle pool, decreased spindle aberrations, and decreased chromosome misalignments, which together culminated in increased litter sizes when compared to nontreated females of the same age [19]. Notably, telomerase activity was elevated and telomere length was also preserved in resveratrol-treated mice [19]. Liu et al. were also able to delay ovarian ageing with short-term treatment (2 months) of the antioxidant N-acetyl-L-cysteine (NAC), reporting an increase in zygote quality and early embryo development as well as increased telomerase activity and longer telomere length. Furthermore, long-term (12 month) administration of low doses of NAC also resulted in increased litter size [218]. Complementing these promising data, mice fed a diet supplemented with antioxidants, vitamins C and E, from weaning onwards also experienced a significant decrease in age-related aneuploidy in their oocytes [219]. Two additional studies from this group also served to demonstrate that either early-onset (from weaning) or late-onset (from 22 to 33 weeks) oral administration of a combinatorial treatment of pharmacological doses of vitamins C and E was successful in ameliorating age-associated decrease in oocyte quantity and quality. Indeed, both timing regimens resulted in increase in ovulated eggs and normal chromosomal alignment at MII [220, 221]. Conversely, experimental mouse models of accelerated ageing (e.g., achieved through the administration of D-galactose) have also shown improvement upon treatment with radical-scavenging agents such as the biliprotein C-phycocyanin [222]. In this context, phycocyanin treatment led to decreased oocyte fragmentation and aneuploidy relative to that of females receiving D-galactose only. The phycocyanin-treated animals also responded with elevating levels of SOD in oocytes such that they were indistinguishable from those levels found untreated control females [222].

In a clinical context, supplementation of the antioxidant melatonin during ovarian stimulation in women undergoing IVF resulted in efficient accumulation into follicular fluid and improved oocyte and embryo quality in aged women [223]. In mice, the long-term dietary supplementation of melatonin, between 10 and 43 weeks of age, was also able to mitigate the phenotype of the ageing oocyte with the restoration of oocyte numbers, fertilisation rate, and blastulation rate. At a molecular level, melatonin largely preserved the mRNA reserve of aged oocytes and stimulated SIRT expression, antioxidant capacity, and ribosome function and maintained telomere length [224]. Similar beneficial effects have also been demonstrated in a bovine model, with the addition of melatonin during bovine IVF and embryo culture promoting blastocyst quality and yield [225].

Notwithstanding these positive outcomes, it is important to note that high doses of antioxidant therapy can have negative effects on female fertility. For instance, Tarin et al. revealed the risks associated with the pharmacological doses of antioxidant required to ameliorate the effects of ageing on oocyte quality. In this study, administration of vitamins C and E had a negative impact on uterine function and resulted in decreased litter sizes, reduced frequency of litters, and poor survival of male pups. The authors attributed these adverse outcomes to an inability of the corpus luteum to support pregnancy, thus leading to an increase in foetal resorption [226]. Additionally, high concentrations of eicosatetraynoic and eicosatriynoic acids, as well as lipoxygenase inhibitors, can reversibly block GVBD in mouse oocytes through their antioxidant action [227, 228]. Furthermore, administration of broad-spectrum ROS scavengers into the ovarian bursa have been shown to significantly decrease ovulation rates in hormonally stimulated mice. This response is apparently mediated by inhibition of LH driven upregulation of ovulation related genes [46]. This collective evidence showcases the delicate balance of pro- and antioxidants required for normal ovarian function and highlights the need for careful screening of safe and efficient antioxidant treatments before such a strategy can be reasonably transferred into a clinical setting.

### 5.2. Mitochondrial Replacement Therapy

In recent years, several groups have advocated for age-related infertility to be treated with mitochondrial replacement therapy (MRT). MRT has, as recently as December 2016, been legally approved in the UK for the treatment of mitochondrial disease [229]. The two most well-studied techniques include maternal spindle transfer (MST) and pronuclear transfer (PNT) [229, 230]. Briefly, MST involves the removal of the spindle-chromosome complex of a donor MII stage oocyte and its replacement with the spindle contents of the intended mother prior to fertilisation. In contrast, PNT involves the removal of the female and male pronuclei from the donor oocyte following fertilisation and before pronuclei fusion has occurred. The pronuclei are then replaced with those of the intended parents.

Considering that aneuploidy at the MII phase of oocyte development is a primary source of age-related infertility, it is apparent that MRT using already segregated nuclear material from oocytes at MII or beyond would not be sufficient in overcoming this obstacle. As such, the benefit of similar techniques for the treatment of age-related infertility remains an active area of research. Indeed, germinal vesicle transfer (GVT) is one promising development whereby the cytoplasmic contents of the oocyte are replaced before the onset of chromosome segregation. GVT involves the removal and replacement of the entire nuclear content including diploid chromosome sets as well as the surrounding nuclear material from GV stage oocytes [231]. A recent study by Nakagawa and FitzHarris was able to demonstrate the value of this technique with the restoration of spindle dynamics in the aged oocyte following reciprocal transfer of nuclei between young and aged GV mouse oocytes [232]. Encouragingly, the efficacy of this technique appears to be conserved in human oocytes as demonstrated in a preclinical study where reconstructed oocytes successfully overcome age associated aneuploidy with a euploidy rate of 80% [233]. However, the authors of this study did offer the cautionary note that the results were based on small oocyte numbers and thus argued for more research in this field. In an alternative study, the GV from young and aged human oocytes were reciprocally transplanted into ooplasts from the opposing age group [234]. Remarkably, 71% of aged GV to young ooplast transfers displayed a normal karyotype compared to only 25% of young GV to aged ooplast transfers [234]. Such findings indicate that the cytoplasmic content of the young oocyte contains the factors necessary to reduce damage in aged oocytes. Furthermore, developmental and fertilisation potential can be restored when the GV of mitochondrially damaged oocytes are transferred into donor ooplasts [87]. Given the electron leakage associated with mitochondrial damage, it could be assumed that the oxidative burden is reduced upon GVT of aged oocytes into young ooplasts and leads us to once again highlight the contribution of OS to the age-related deterioration of the oocyte. Despite these encouraging results, considerable research is still required before MRT is likely to receive widespread adoption as a therapeutic intervention of choice to treat age-related female infertility.

### 5.3. Calorie Restriction

Dietary caloric restriction (CR) in adult life could be an alternate mechanism to reduce OS within the oocytes of older women. Calorie restriction has been linked to a decrease in free radical production by the mitochondria [1, 235–237]. After as little as 6 weeks of 40% CR in rats, ROS production was reduced by 45% and mtDNA damage by 30% in cardiac cells [238]. While translation into nonhuman primates has been controversial, there is evidence to suggest that CR also delays the onset of age-related disorders in rhesus monkeys [239]. Moreover, the application of the same CR regimen for an extended period of 7.5 months was shown to maintain oocyte quality in 12-month-old mice. Indeed, oocytes from the 12-month-old CR mice presented with significantly decreased aneuploidy, meiotic spindle abnormalities, mitochondrial dysfunction, and chromosomal misalignment compared to noncalorie-restricted age-matched control animals [1]. In seeking to account for this positive effect, the authors posited that it was likely attributed to a decrease in the expression of peroxisome proliferator-activated receptor *γ* coactivator-1*α* (PGC-1*α*), a transcriptional regulator of several genes associated with mitochondrial respiration. It is therefore possible that reduction in mitochondrial-derived ROS mediated by CR in adult life could be an effective means of improving the fertility of women of advancing maternal age. However, this study was performed for over 40% of the animal's lifetime and therefore a more practical model would need to be developed for human use.

## 6. Concluding Remarks

The causative relationship between the age-related decline in oocyte quality and increases in oxidative stress (OS) is well accepted. A concomitant decline in the fidelity of the protective mechanisms that oocytes are capable of mounting to mitigate oxidative insults only acts to perpetuate the intensity of the oxidative damage. Indeed, an attenuation of the efficacy of ROS metabolism, DNA repair, spindle assembly checkpoint, capacity for protein repair, and/or degradation collectively render the aged oocyte acutely vulnerable to oxidative insult. While antioxidant treatments, mitochondrial replacement therapy, and calorie restriction offer the potential for therapeutic intervention for the treatment of age-related infertility in women of advanced maternal age, it is clear that each of these strategies would benefit from further refinement before their full potential can be realised. In this review, we conclude that the accumulation of oxidative damage in the maternally aged oocyte likely results from the convergence of an increase in ROS production as well as a decreased capacity of the oocyte to mitigate oxidative damage. We also urge further basic research into therapeutic interventions to enhance the activity of oxidative repair pathways in the oocyte or otherwise ameliorate the damaging impact of OS lesions in these cells.

## Figures and Tables

**Table 1:** Alterations in gene expression of pathways involved in mitigating oxidative damage in the aged oocyte.

Category	Genes	Cellular compartment	References
Antioxidants	↓*Sod1*, ↓*Sod2*, and ↓*Cat*	Human granulosa cells	[112]
	↑*Gpx1*, ↓*Gstm2*, ↓*Prdx3*, and ↓*Txn2*	Mouse ovaries	[13]

	↓*Sod1*, ↓*Txn1*, ↓Txndc9, and ↓*Apacd*	Mouse MII oocytes	[97]
	↓*Apacd*, ↓*Glrx*, ↓*N33*, *↓Pdcl*, *↓Grp58*, and *↓Pdia6*	Human MII oocytes	[96]
	*↓Txnrd1 and ↓ Sod1*	Mouse GV oocytes	[3]
↓*Txnrd1*, ↓*Txnrd3*, and ↑*Sod2*	Mouse MII oocytes	[3]
			

Sirtuin proteins	↑*Sirt1*	Mouse GV oocyte	[127]
	↓*Sirt3*	Human granulosa and cumulus cells	[133]
	↓*Sirt2* and ↓*Sirt6*	Mouse cumulus cells	[136]
			

DNA repair/checkpoint	↓*Brca1*, ↓*Mre11*, ↓*ATM*, and ↓*Rad51*	Mice and human primordial follicles and GV oocytes	[142]
	↓*Brca1*	Mouse MII oocytes	[3]
	↓*Atr*, ↓ *Chek1*, ↓*Nbs1*, and ↓*Rad17*	Human MII oocytes	[96]
	↓*Tert*	Mouse MII oocytes	[148]
			

Ubiquitin proteasome system	*↓Psmb2*, *↓Psmb5*, *↓Psmc2*, *↓Psmd4*, *↓Psmd8*, *↓Ubap1*, *↑Ube2h*, *↑Usp15*, *↓Usp2*, *↓Usp31*, *↑Usp7*, and *↑Usp8*	Mouse GV oocytes	[3]
	*↑Psma4*, *↑Psmb4*, *↑Psmc2*, *↓Psmc5*, *↓Psmd9*, *↑Psmd11*, *↓Psme3*, *↓Psmf1*, *↑Ubap1*, *↓Ube1c*, *↑Ubc*, *↑Ube2a*, *↓Ube2d1*, *↓Ube2d2*, *↓Ube2h*, *↓Ube3a*, *↑Usp15*, and *↑Usp8*	Mouse MII oocytes	[3]
	*↓Anapc4*, *↓Hip2*, *↓Ubc*, *↓Ube1c*, *↓Ube2a*, *↓Ube2e3*, *↓Ube2g1*, *↓Usp1*, *↓Usp30*, *↓Psma6*, *↓Psmb1*, *↓Psmb4*, *↓Psmc2*, *↓Psmc3*, *↓Psmd12*, and *↓Siah2*	Mouse MII oocytes	[97]
	*↓Hip2*, *↓Psmc2*, *↓Psmc6*, *↑Psmd2*, *↑Psmd5*, *↑Psmd1*, *↑Psmd9*, *↑Ubch9*, *↓Ube2n*, *↓Ube2e*, *↓Ube2g1*, *↑Ube2h*, *↑Ube2v*, *↑Ube3a*, *↓Usp1*, *↓Usp8*, and *↓Usp9x*	Human MII oocytes	[96]
			

Chaperones	*↓Cct2*, *↓Cct3*, *↓Cct5*, *↓Tra1*, *↓Dnaj1*, *↓Hsp86-1*, *↓Hspa4*, *↓Hsp70-4*, *↓Hspa8*, *↓Vbp1*, *↓Mmp2*, *↓Skt25*, *↓Map4k5*, and *↓Ndr4*	Mouse MII oocytes	[97]
	*↓Cct2*, *↓Dnajb1*, *↓Dnajb6*, *↓Dnajc5*, *↓Hspa14*, *↓Hspa1b*, *↓Hspa8*, *and ↓Hspcb*	Mouse GV oocytes	[3]
	*↑Cct2*, *↓Cct7*, *↑Dnaja1*, *↓Dnaja2*, *↓Dnaja4*, *↓Dnajb10*, *↓Dnajb11*, *↓Dnajc3*, *↓Dnajc8*, *↓Hspa1b*, *↓Hspa1b*, *↓Hspa9a*, *↑Hspb1*, *↓Hspcb*, *and ↓Mmp2*	Mouse MII oocytes	[3]

**Table 2:** Alterations in protein expression and activity of pathways involved in mitigating oxidative damage in the aged oocyte.

Category	Proteins/hormones	Cellular compartment	References
Antioxidants	↓SOD1, ↓SOD2, and ↓CAT	Human granulosa cells	[112]
	↓SOD1 and ↓SOD (activity)	Human cumulus cells	[113]
	↓GST (activity and expression), ↓CAT (activity), and ↑SOD (activity)	Human follicular fluid	[114]
	↓GST (activity), ↓GR (activity), and ↓GPX (activity)	Human follicular fluid	[24]
	↓SOD and ↓GPX	Human ovaries	[115]
	↓TXN1 and ↓GSTM5	Mouse MII oocytes	[116]
	↓GST (activity)	Mouse MII oocytes	[117]
	↓Melatonin	Human follicular fluid	[121]
Sirtuin proteins	↓SIRT1	Mouse GV oocyte	[127]
	↓SIRT2	Mouse MII oocyte	[137]
	↓SIRT3	Human granulosa and cumulus	[133]
DNA repair/checkpoint	↓Telomerase (activity)	Human ovary	[147]
	↓Telomerase (activity) and ↓TERT	Mouse MII oocytes	[148]
UPS	↓PSMD12 and ↓USP15	Mouse MII oocytes	[116]
Chaperones	*↑*Dnajc19 and *↑*Dnajc11	Mouse MII oocytes	[116]
